# Ever dispense of prescribed allergy medication in children growing up close to traffic: a registry-based birth cohort

**DOI:** 10.1186/s12889-015-2356-3

**Published:** 2015-10-06

**Authors:** Anna Lindgren, Emilie Stroh, Kristina Jakobsson

**Affiliations:** Division of Occupational and Environmental Medicine, Lund University, SE-221 85 Lund, Sweden

**Keywords:** Air pollution, Allergy, Children, NOx, Traffic, Allergy medication, Antihistamines, Nasal corticosteroids

## Abstract

**Background:**

Epidemiologic studies have shown conflicting results regarding the role of traffic pollution in the development of allergic disease. This study investigated the relationship between living close to traffic and ever dispense of prescribed oral antihistamines or nasal anti-allergic medication, among young children. The underlying aim was to investigate if children growing up close to traffic pollution are at higher risk of developing allergy in early childhood.

**Methods:**

We investigated a birth cohort in southern Sweden, consisting of *N* = 26 128 children (0–6 years) with health outcome and exposure data. Of these children, *N* = 7898, had additional covariate information. Traffic intensity and yearly averages of dispersion-modeled concentrations of NO_X_ (100 × 100 m grid) at residential addresses, were linked with registry data on dispensed allergy medication (the Swedish Prescribed Drug Register). Individual level covariate information was obtained from questionnaires distributed to parents at Child Health Care-center visits, eight months after birth. Cox proportional hazards regression was used for the statistical analyses.

**Results:**

Living in close proximity to a road with equal to or greater than 8640 cars/day (compared to 0–8639 cars/day), was not associated with higher incidence of ever dispensed oral antihistamine or nasal anti-allergic medication, with or without adjustment for confounders (sex, breastfeeding, parental allergy, parental origin, season, and year of birth). Similar results were found in relation to NO_X_.

**Conclusions:**

Traffic-related exposure was not associated with higher incidence of ever dispensed medication against allergy, in children 0–6 years in southern Sweden. These results indicates that traffic-related exposure may not be a risk factor for early onset allergy in children in southern Sweden. However, children with dispense of prescribed allergy medication may be a selected subgroup, and the results for this group may not be generalizable to all children with allergy.

**Electronic supplementary material:**

The online version of this article (doi:10.1186/s12889-015-2356-3) contains supplementary material, which is available to authorized users.

## Background

Experimental studies suggests that air pollution from traffic can cause or enhance allergic symptoms [1], but epidemiological studies are more inconsistent [2–4].

In childhood, allergic disease often develops in a predictable way in individuals with an allergic predisposition, “the allergic march”. This starts with eczema and food allergy in early childhood, followed by a higher risk of wheeze and infectious asthma in pre-school age, and allergic asthma and allergic rhinitis in subsequent age [5]. Allergic rhinitis is uncommon in early childhood, but the prevalence increases in preschool and school age [6]. The natural history of asthma and its relation to traffic has been discussed in a previous article [7].

The main drug used for allergic symptoms are oral antihistamines, which have systemic anti-allergic effect, and are prescribed for food allergy symptoms, such as urticaria, and for allergic rhinitis. An alternative or complementary treatment for allergic rhinitis is locally inhaled nasal corticosteroids/other nasal anti-allergics which are used mainly for allergic rhinitis. Oral antihistamines and nasal corticosteroids are often prescribed by doctors, but can also be bought over the counter, without a prescription. However, a doctor prescription is more likely to be specific for allergic symptoms, since clinical examination/anamnesis excludes infectious rhinitis as a cause. Some studies have used purchase of inhaled oral antihistamines/ nasal corticosteroids as a proxy for seasonal allergic rhinitis incidence or prevalence [8].

The Swedish Prescribed Drug Register has a complete (99.7 %) coverage of individual-level dispensed medication for all individuals living in Sweden [9], and dispensed allergy medication will in this study be used as a proxy variable for incidence of allergy. Dispersion-modelled levels of outdoor Nitrogen Oxides (NO_X_) and data on traffic intensity residential addresses were used to estimate children’s exposure to traffic pollution. The underlying aim was to investigate if children growing up close to traffic pollution are at higher risk of developing allergy in early childhood.

## Methods

### Study area

Scania is the southernmost county of Sweden, with a population of 1 243 329, in year 2010 [10]. Children born in Scania, whose mothers were registered as living in one of the municipalities Malmö, Svedala, Vellinge and Trelleborg were included, since survey data with covariate information were available from Child Health Care centers (CHC) in these areas.

Malmö has the highest level of air pollution in the area. Although pollutant levels in the region are low in a European context, they are higher than in most of Sweden, due to long-range transport of pollutants from the continent and extensive transport/road, harbor and ferry traffic. The study area has been described in detail previously [11, 12].

### Selection of study population

**Fig. 1:**
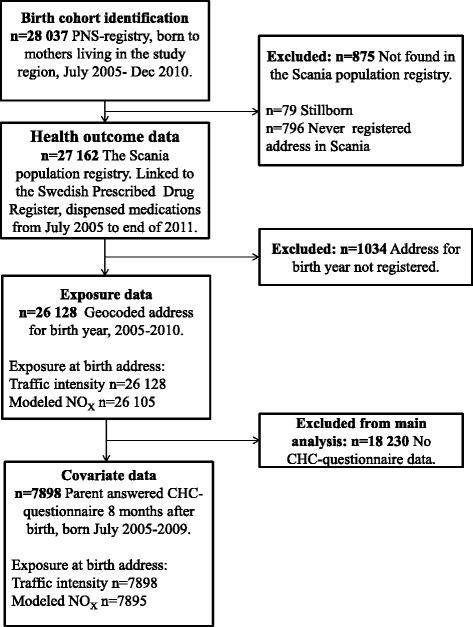
Selection of study population

A flow-chart of the study population selection is displayed in Fig. 1. The study was limited in time to children born from July 2005, since individual level medication data is only available since then. All children were followed with health outcome data to the end of 2011.

To identify a birth cohort, we retrospectively retrieved the identity number of all children born by mothers living in Malmö, Svedala, Vellinge and Trelleborg during July 2005- Dec 2010, from the Perinatal Revision South (PNS)-registry. The PNS registry has 100 % coverage of visits on obstetric and peri/prenatal units in the county. Out of 28 037 children identified in the PNS-register, 875 were not found in the Scania population registry and thus excluded, since they were not registered as living in the region during childhood. Health outcome data was available for all children found in the Scania population registry (*n* = 27 162), by linkage to the Swedish Prescribed Drug Register. Geographical coordinates (geocodes) for registered birth year address was available for 26 128 children, for which exposure was assessed. Most of the missing geocodes belonged to children born in December, whose late birth date probably lead to addresses not being registered during year of birth. Geocodes were retrieved for birth year and subsequent years for each child, until the end of 2010. Finally, covariate information from questionnaires routinely distributed at Child Health Care centers was available for 7898 children, which formed the main study cohort.

### Ethical permission

This study was approved by the Lund University Ethical Committee (registration no. 2011/468). No formal informed consent was required, but the study was advertised in the local newspaper and information was distributed to Child Health Care centers, allowing parents to request that their children not be included in it. No such request was raised.

### Allergy medication

The Swedish Prescribed Drug Register includes all drugs dispensed at pharmacies in Sweden, since July 2005 linked to personal identity numbers [13]. The registry is maintained by the National Board of Health and Welfare. All expedited drugs on the pharmacies are registered, with a very small number of incorrect or incomplete registrations of ID. The population coverage with correct patient identities is 99.7 % [9].

The registry contains data on all dispensed prescriptions in ambulatory care. Over-the-counter (OTC) medications and drugs used at in-patients settings are not included. Medication data are classified according to the Anatomical Therapeutic Chemical (ATC) Classification System [14].

The Pharmaceutical Benefit Scheme, which is mainly tax financed, covers the main costs for drugs in ambulatory care in Sweden. There is a ceiling on the total amount that a patient pays during a 12-month period for subsidized pharmaceuticals (2013: SEK 2200, €252). The drug costs of children younger than 18 years, living in the same household, are counted together.

We obtained information on medications prescribed for allergy. The health outcomes used were dispensed prescription of oral antihistamines (ATC-codes: R06), nasal corticosteroids (ATC-codes: R01AD), and nasal anti-allergic agents excl. corticosteroids (R01AC).

As primary health outcomes we used:

1. Incidence of first ever dispensed oral antihistamine
2. Incidence of first ever dispensed nasal corticosteroid/other nasal anti-allergics

First dispensed oral antihistamine was seen as a proxy for incidence of allergy, but is non-specific. First dispensed nasal corticosteroid/nasal anti-allergics was seen as a proxy for incidence of allergic rhinitis.

### Exposure assessment

Geocodes for the children’s officially registered residential addresses were retrieved from the population registry for each year from birth until the end of 2010. Although health outcome data are available until end of 2011, the exposure data are thus only available until end of 2010. Individuals are geographically positioned at the center coordinate of their residence.

### Traffic intensity

A Geographical Information System (GIS) based registry from the Swedish National Road Database, provided data on traffic intensity in all major roads in the county. To assess exposure to traffic, we identified the road with the heaviest traffic intensity within 100 m of the residence. Traffic intensity was categorized as “no road”, “road with 0–2879 cars/day”, “2880–8639 cars/day”, “8640–14,400 cars/day”, and “≥14,400 cars/day”, based upon daily (24-hour) mean levels.

The traffic intensity categories were merged into a dichotomous variable, “0–8639 cars/day” (including children with “no road”) and “≥8640 cars/day”, to obtain enough power, since not enough cases lived in the highest exposure category to assess it separately. The classification was based upon results from previous studies in the same geographical region, which found a higher prevalence of asthma among adults living within 100 m of roads with ≥8640 cars/day [15, 16]. Separate analyses were done in relation to traffic intensity for: 1) birth address exposure 2) birth address exposure, with children censored when/if they moved during time at risk.

### Modeled concentrations of NO_X_

Concentrations of NO_X_ (NO_2_ + NO) at each child’s residential address were modeled as annual means for each calendar year 2005–2010, with a spatial resolution of 100 × 100 m. This has previously been described in detail [12]. Validation of this model with a resolution of 100 × 100 m has shown satisfying agreement between modeled and measured concentrations of NO_2_ (Spearman’s *r* = 0.8) [17].

Separate NO_X_ -analyses were done for: 1) birth address exposure 2) birth address exposure, with children censored when/if they moved during time at risk, and 3) mean NO_X_ during all years at risk (excluding 2011 for which geocodes were not available). The mean NO_X_ during time at risk was only assessed for those never moving outside the study area during time at risk. Since time at risk differ with health outcome, the number with modeled mean NO_X_ during time at risk, also vary depending on health outcome.

We used a categorical classification of NO_X,_ since previous studies in the same geographical region have indicated non-linear relationship between outdoor NO_X_ and respiratory disease [15, 16]. We based our categories on exposure contrasts (≤15, 15–25 and >25 μg/m^3^), rather than on the distribution of NO_X_ in the population.

### Covariate information

The final cohort for the main analysis included 7898 children born in the region, whose parents had answered a CHC center questionnaire 8 months after birth. The questionnaire was handed out to parents in Malmö, Svedala, Vellinge and Trelleborg, in conjunction with their children’s 8 month checkup at the CHC centers [18]. The questionnaire had been validated and translated from Swedish into five different languages: Albanian, Arabic, English, Serbo-Croatian, and Somali. The response rate varied between years but was approximately 65 % of handed out questionnaires [18].

Variables considered for inclusion in the multivariable models were: sex, birth weight, smoking during pregnancy, environmental tobacco smoke (ETS), mold at home, parental allergy, furred pets at home, breastfeeding, parental origin, parental education, problems to pay bills, type of housing, season, and birth year.

### Statistics

All statistical analyses were performed using SAS, version 9.3. Survival analysis was performed because of different lengths of follow-up of the children. We used the same analysis plan as in a previous article on childhood respiratory disease [7].

We used two different censoring variables: 1) children were censored at year of study end (2011), or 2) children were censored when they moved from their original birth address, or at year of study end (2011). Descriptive Kaplan-Meier survival curves, with numbers at risk, were displayed for all health outcomes. The proportional hazard assumptions for exposure and health outcome were checked graphically by log(−log(survival))-curves, and these were approximately fulfilled for the larger cohort (*n* = 26 128), but not for all health outcomes in the smaller main sample (*n* = 7898) which was expected since the smaller cohort is less numerically stable.

We then reported unadjusted Cox Proportional Hazards-ratios (Cox PH) between exposure and health outcomes. We used prescreening of variables in combination with a stepwise Cox PH-procedure, to select covariates to include in the final multivariable models. We performed the same selection procedure for all health outcomes in relation to traffic intensity, to find the most important predictors. Any variable staying in any of the health outcome models, was included in all the models, for model consistency. Traffic intensity was forced to remain in the model in each step. The following steps were done:

1. Univariable prescreening of all covariates in Table 1, except municipality. Any variable with a univariate p-value <0.2 for the hazard ratios (HR) between the covariate and the health outcome, was selected to next step.

**Table 1:** Description of the main cohort, *n* = 7898

		N (%)	HR (95 % CI)^a^	
			Nasal anti-allergics	Oral antihistamine
			1st purchase	1st purchase
Sex	Girl	3784 (49)	1.0	1.0
	Boy	3996 (51)	1.40 (1.04–1.90)	1.03 (0.95–1.12)
	Missing	118		
Birth weight	2500–4000 (normal)	6079 (78)	1.0	1.0
	500–2499 (low)	301 (4)	1.38 (0.70–2.71)	0.98 (0.78–1.22)
	4001–6500 (high)	1396 (18)	0.95 (0.64–1.41)	1.02 (0.91–1.13)
	Missing	122		
Smoking during pregnancy	No	7275 (94)	1.0	1.0
	Yes	499 (6)	1.07 (0.61–1.88)	1.08 (0.92–1.27)
	Missing	124		
Environmental tobacco smoke	No	6591 (85)	1.0	1.0
	Yes	1177 (15)	0.89 (0.58–1.36)	0.97 (0.86–1.09)
	Missing	130		
Breastfeeding	≥8 months	3920 (56)	1.0	1.0
	<8 months	2807 (40)	0.91 (0.67–1.24)	1.19 (1.09–1.30)
	Never breastfed	278 (4)	0.79 (0.32–1.95)	1.08 (0.86–1.36)
	Missing	893		
Parental allergy	No	3177 (46)	1.0	1.0
	Yes	3751 (54)	1.18 (0.86–1.62)	1.14 (1.05–1.25)
	Missing	970		
Furred pets at home	No	5790 (75)	1.0	1.0
	Yes	1922 (25)	1.12 (0.80–1.56)	1.13 (1.03–1.25)
	Missing	186		
Mold at home	No	7326 (95)	1.0	1.0
	Yes	386 (5)	1.26 (0.69–2.32)	0.99 (0.81–1.20)
	Missing	186		
Problems to pay bills	Never or seldom	7361 (96)	1.0	1.0
	Yes, >6 months/year	348 (5)	0.84 (0.40–1.80)	0.92 (0.75–1.14)
	Missing	189		
Swedish parents	Yes, both Swedish	297 (4)	1	1.0
	One foreign	1792 (23)	1.03 (0.68–1.55)	0.93 (0.83–1.04)
	Both foreign	5612 (73)	1.16 (0.81–1.66)	0.80 (0.72–0.89)
	Missing	197		
Highest education any parent	>12 years	297 (4)	1.0	1.0
	10–12 years	1792 (23)	1.14 (0.48–2.68)	1.13 (0.89–1.44)
	≤9 years	5612 (73)	1.22 (0.54–2.76)	1.16 (0.92–1.47)
	Missing			
Type of housing	Owned house	2384 (36)	1.0	1.0
	Tenant-owned apartment	2242 (29)	0.69 (0.47–1.01)	0.93 (0.84–1.03)
	Rented apartment	2616 (34)	0.84 (0.60–1.18)	0.86 (0.78–0.95)
	Other	101 (1)	---	1.14 (0.80–1.61)
	Missing	156		
Municipality	Vellinge	449 (6)	1.0	1.0
	Svedala	664 (8)	0.70 (0.35–1.42)	1.25 (1.01–1.53)
	Trelleborg	611 (8)	0.65 (0.39–1.08)	0.81 (0.69–0.96)
	Malmö	6134 (78)	0.65 (0.32–1.32)	1.01 (0.82–1.25)
	Missing	40		
Birth year	2005	1066 (14)	1.0	1.0
	2006	2395 (30)	1.94 (1.18–3.19)	1.22 (1.07–1.39)
	2007	1664 (21)	1.66 (0.90–3.04)	1.10 (0.95–1.28)
	2008	2179 (28)	2.56 (1.34–4.90)	1.17 (1.01–1.35)
	2009	594 (8)	2.44 (0.68–8.77)	1.48 (1.19–1.84)
Birth season	Winter (Dec–Feb)	1957 (25)	1.0	1
	Spring (March–May)	1946 (25)	1.12 (0.76–1.67)	1.02 (0.91–1.15)
	Summer (June–Aug)	1835 (23)	0.47 (0.30–0.73)	0.85 (0.75–0.95)
	Autumn (Sep–Nov)	2160 (27)	0.59 (0.40–0.88)	0.87 (0.78–0.98)

HR (95 % CI) for dispense of medication in relation to population characteristics, for people who may have moved during study time

^a^Unadjusted --- Too few individuals, numerically unstable results
2. All the selected variables were included into a multivariable Cox PH model, together with traffic intensity which was forced to stay in the model. Backward selection was performed, with significance level for staying at 0.1.
3. Starting with an initial model including the variables selected from step 2. Forward selection was performed, with significance level for entry at 0.2, to consider for inclusion the variables initially not selected at step 1.
4. Starting with the model selected from step 3. Finetuning was done by stepwise selection- entry/staying at 0.05. The variables selected to be included in the final multivariable models were: Sex, breastfeeding, parental allergy, parental origin, season, and year of birth.

All selected covariates except year of birth approximately fulfilled the proportional hazard-assumption, and we used the Cox PH model for the final multivariable analyses, to assess the incidence of asthma medication and diagnoses in relation to traffic-related exposures. Multivariable analyses presented do not include children with missing values for any of the variables included.

We also performed sensitivity analyses: we analyzed the unadjusted relation between traffic-related exposure and health outcomes, for all children with complete information on exposure and health outcome (*n* = 26 128). For the main cohort (*n* = 7898), we separately estimated effects for Malmö vs. the remaining study area, to see if results were consistent across geographical regions. We also performed analyses restricted to children with high socio-economic status (*n* = 3464), here defined as children whose parents fulfilled all the following criteria; never problems to pay bills, at least one parent with >12 years education, and both parents born in Sweden. The question about ability to pay bills was here not dichotomized as in the main analysis, but instead a finer original classification was used, where “never problems to pay bills” was separated from “seldom problems to pay bills”.

Finally, as a robustness test, we performed an analysis for a model including all possibly relevant covariates, to see if the results would differ from the stepwise model and the unadjusted model. We also display model fit measured by Akaike’s Information Criteria (AIC), for the fully adjusted, the stepwise, and the unadjusted model, based on individuals with complete covariate information data (*n* = 5736).

The HR in all analyses was displayed with 95 % confidence intervals (CI).

## Results

### Covariate description

Population characteristics, and incidence of allergy medication in relation to these characteristics, are displayed in Table 1. Oral antihistamines were dispensed in a higher proportion of children breastfed less than 8 months, with parental allergy, with furred pets at home, or for those living in Svedala or born certain years (2006, 2008, 2009). Nasal anti-allergics were dispensed in a higher proportion of boys than girls and for children born certain years (2006, 2008).

### Missing data and Kaplan-Meier survival curves

**Fig. 2:**
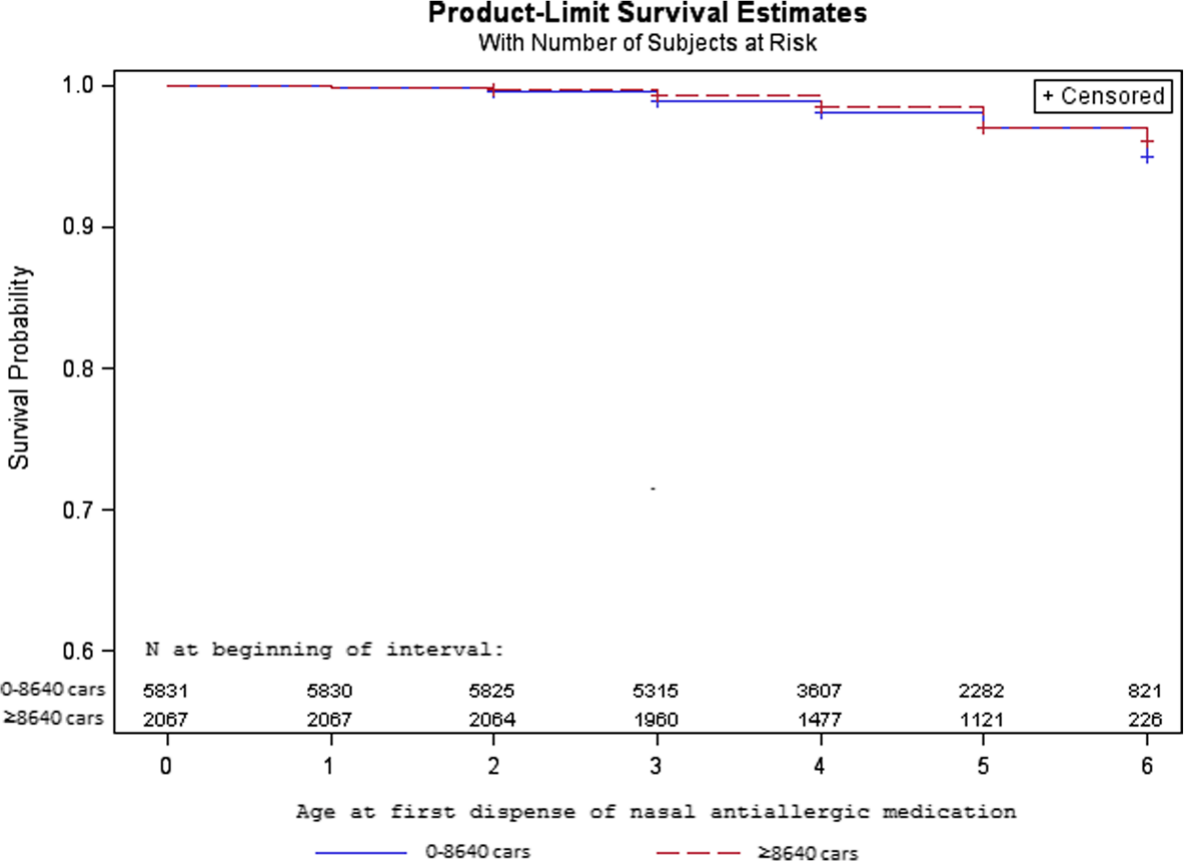
Kaplan-Meier survival curves of nasal anti-allergic medication, by traffic intensity

**Fig. 3:**
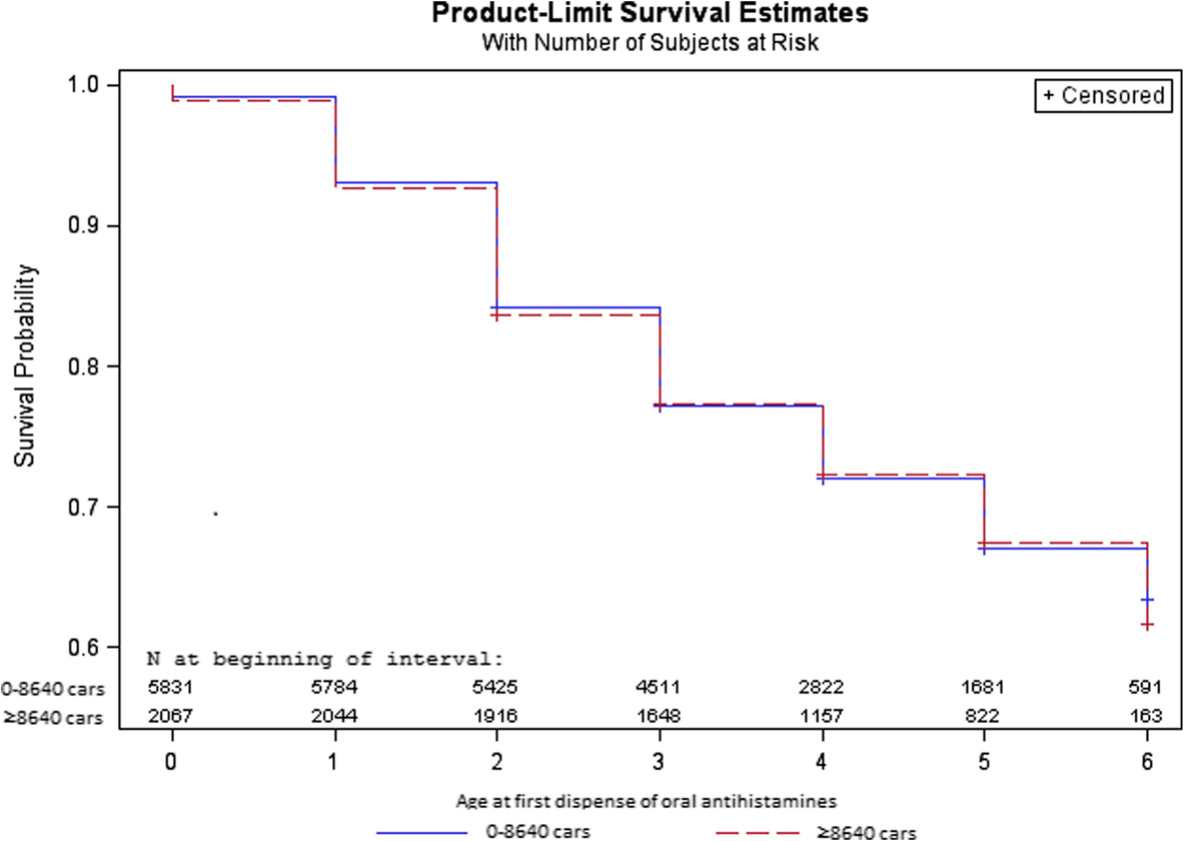
Kaplan-Meier survival curves of oral antihistamines, by traffic intensity

The Kaplan-Meier survival curves (Figs. 2 and 3) showed almost no incidence for nasal anti-allergic medication before the age of 2 years, and then increasing dispense until age 6. For oral antihistamines, the incidence of dispense was more evenly spread from age 0 to 6.

### Incidence of dispensed allergy medication in relation to traffic

There was no association between living close to traffic and incidence of dispensed nasal anti-allergic medication or oral antihistamines [Table 2]. Similar results were seen in relation to NO_X_ for nasal anti-allergic medication [Table 2]. For oral antihistamines, living in areas with high NO_X_-levels was statistically significantly associated with a lower incidence of dispense of oral antihistamines [Table 2].

**Table 2:** Adjusted HR (95 % CI) for allergy medication, in relation to traffic-related exposure, *n* = 7898

Allergy medication^a^		
	Nasal anti-allergics	Oral antihistamine
	1st purchase	1st purchase
Heaviest road ≤100 m, birth address^b^		
0–8639 cars/day	1.0	1.0
≥ 8640	0.89 (0.61–1.29)	0.91 (0.83–1.00)
Heaviest road ≤100 m, never moved^b^		
0–8639 cars/day	1.0	1.0
≥ 8640	0.83 (0.51–1.36)	0.90 (0.79–1.03)
NO_X,_ birth address^c^		
≤ 15 μg/m^3^	1.0	1.0
15–25	0.84 (0.58–1.21)	0.86 (0.77–0.95)
> 25	0.86 (0.50–1.49)	0.81 (0.68–0.96)
NO_X_, never moved^c^		
≤ 15 μg/m^3^	1.0	1.0
15–25	0.95 (0.62–1.47)	0.88 (0.78–0.99)
> 25	0.95 (0.42–2.16)	0.77 (0.62–0.96)
NO_X,_ lifetime mean^d^		
≤ 15 μg/m^3^	1.0	1.0
15–25	0.92 (0.62–1.35)	0.84 (0.75–0.93)
> 25	0.59 (0.23–1.50)	0.70 (0.56–0.89)

^a^Adjusted for sex, season, parental origin, year of birth, breastfeeding, and parental allergy

^b^ *n* = 6093 children had complete covariate information and traffic exposure assessments

^c^ *n* = 6091 children had complete covariate information and modeled NOx concentrations

^d^The number of children with complete covariate information and modeled mean NOx during time at risk, was *n* = 5264 for nasal antiallergics, and *n* = 5317 for oral antihistamines

### Sensitivity analyses

An analysis of all children for which health outcome and exposure data were available (*n* = 26 128), unadjusted for any factors, showed that living close to a road with high traffic, or in areas with high level of NOx, was not associated with incidence of dispensed nasal allergic medicine. However, it was statistically significantly associated with a lower incidence of dispense of oral antihistamines (Additional file 1: Table S1).

For the main cohort (children with covariate information, *n* = 7898), we stratified our analyses separately for Malmö vs. the remaining municipalities, and the results were largely consistent across areas (Additional file 1: Table S2).

We also performed an analysis restricted to children with high socio-economic status (*n* = 3464) and the results for this subgroup were similar to the results for the main cohort (Additional file 1: Table S3).

Finally, as a robustness test, we performed an analysis for a model including all possibly relevant covariates (“fully adjusted model”) and the results were similar to the results for the stepwise and unadjusted model (Additional file 1: Table S4). The model fit, measured by AIC, was consistently better for the stepwise model for oral antihistamines, and largely better for the stepwise mode for nasal antiallergics, compared to the full and the unadjusted models.

## Discussion

There was no increased risk of ever dispense of prescribed allergy medication among children 0–6 years, growing up close to a road with high traffic intensity, or with high levels of outdoor NO_X_ at residence.

### Strengths and limitations

A strength of the study was the large register-based health outcome data which prevents recall and awareness bias, although other types of biases may exist due to different health-seeking behavior [11]. A strength was that most of these biases can however be reduced since we had confounder information which was used to stratify e.g., on socio-economic status. A limitation was the low power for nasal anti-allergic medication, which was inevitable since this is a rare health outcome in young age. However, that the results for the larger study population, which had more power, were the same as for the cohort with questionnaire information strengthens that there is no higher incidence of allergy in areas with high traffic pollution.

A major strength was also the exposure data in this study. Residential addresses for each year since birth were known, which exclude a migration bias which could otherwise be expected to dilute the effects. We also had validated high quality exposure data for NO_X_, modeled with a high resolution, which further minimize the risk of other exposure misclassification biases which could be expected to dilute the effects. The exposure situation in the area has been described in more detail in a previous article. Mean NO_X_ at birth year was 17 μg/m^3^, and the percentile distribution was 9.2, 11.8, 17.6, 21.1, and 24.6 μg/m^3^ (10th, 25th, 50th, 75th, and 90th percentile). Min, Max = (6.1, 45.9) μg/m^3^ [7].

A limitation was that prescribed dispensed medication does not cover all allergy medication use, since some are bought over the counter without prescription. Children with dispense of prescribed allergy medication may be a highly selected subgroup [11], and the results for this group may not be generalizable to all children with allergy. However, we believe that many parents are likely to go to a doctor when their preschool children have allergic problems, and that a large proportion of young allergic children get allergy prescription compared to if allergy develops in older age when over-the-counter medication may make up a larger proportion. The validity of dispensed allergy medication as a proxy for allergy incidence might thus decrease with age, since it is likely that adults buy over-the-counter medication for e.g., allergic rhinitis without seeing a doctor, and dispensed medication may then represent a small selection with more severe disease. We believe this may become an increasing problem if the cohort is followed after early childhood. A doctor prescription is however likely to be more specific for allergic symptoms than over-the-counter medication, in all age groups, since clinical examination/anamnesis excludes infectious rhinitis as a cause.

It should be noted that the overall prescription of nasal corticosteroids in Scania has been increasing during 2006–2011 [19], something which may explain that children born in 2008–2009 had a higher incidence of nasal anti-allergics than children born earlier in this study. However, this did not affect our results since we adjusted for year of birth. Finally, it should be noted that nasal corticosteroids are not exclusively prescribed for allergic disease, but can also be prescribed e.g. for nasal polyposis.

### Comparison with other epidemiological studies

Overall, epidemiological studies have shown conflicting results of the relation between traffic and allergy. In a review 2009 of the relation between air pollution and sensitization in four birth cohorts, three of the birth cohorts found some association with sensitization, while one did not [4]. However, there was heterogeneity both in what health outcome was associated with air pollution and also in the exposure assessment. In some of the individual studies associations have been found with inhalant exposure such as pollen [20, 21]. In other studies however, associations were mainly found with food allergens [22, 23]. In a meta-analysis by Gruzieva et al. [3], a standardized modelling technique was applied to all the four cohorts, and also a fifth cohort, and this meta-analysis found no overall association between air pollution and allergic sensitization. In a recent meta-analysis by Bowatte et al. [2], of birth cohort studies, they found that increasing exposure to PM 2.5 was associated with sensitization to both aero- and food allergens. There was also some evidence that traffic related air pollution was associated with hay fever [24].

Few have investigated the relation with allergic rhinitis in childhood, but there has been positive association in some cohorts and cross-sectional studies [20, 25–30], while some studies have found no association [31–36]. No other study has used medication data as a proxy for allergy in long-term studies of traffic, although medication data has been associated with short-term effects of pollen [37, 38] and to measure the relation to other risk factors [8].

We have in a previous cross-sectional study found an association with allergic but not non-allergic asthma and rhinitis in adults [16]. We however found no association with asthma medication in childhood in the present cohort [7]. We have therefore hypothesized that there may only be an association between long-term exposure to air pollution and asthma after early childhood.

### Experimental evidence for an association between traffic and allergy

For respiratory outdoor allergens such as birch pollen, it has been suggested that nitration by Nitrogen Dioxide (NO_2_) and Ozone (O_3_) increases allergenicity [39–42]. Antibodies to pollen may cross-react with food allergens, but there is also support that diesel exhaust particles could block oral tolerance mechanisms and thereby contribute to food allergen sensitization *per se* [43].

A general theory with experimental support is that air pollution shifts Th1/Th2 response in the immune system, thereby promoting allergy development of the Ig-E-mediated type [44]. Particles can both act as immunomodulators but also as vectors for respiratory exposure, and also facilitate uptake by antigen-presenting-cells [45, 46].

In summary, there is ample evidence from experimental studies that do support a relation between air pollution and allergy, in a number of different possible ways [1, 47–50]. However, it is also very clear that the development of allergy and sensitization is not simple, and the mechanisms for allergy development are mainly unknown. This can be illustrated by the simple fact that it has still not been established if growing up in a household with furred animals is a risk factor, or a protecting factor, for allergy [51]. No clear dose-relation between allergy development and allergen exposure has been found [52].

### Implications for further studies

Our study adds to the many studies which do not support a clear relation between traffic exposure and allergy development in early childhood. However, some studies find an association. It is obvious that this relationship in case it exists, is not a simple one, and may be overridden by other risk factors.

A complicating factor is that manifest allergy is a combination of genetic factors, allergy-promoting and allergy-protecting factors, and also actual exposure to allergen. The result in this study weakly suggests that people living in areas with high levels of NO_X_ may even develop allergy to a lesser degree then people living in unpolluted areas. It is unlikely that traffic is a protecting factor, but more likely that actual exposure to allergen or other allergen-promoting factors differ. One factor that may differ between urban and rural areas are pollen counts, it has been shown in one study that rural areas had pollen with higher allergenicity, but a lower total level of pollen [53]. Further studies may try to get more data on local variations in pollen counts or birch-intensity in different areas. Further studies of this child cohort in older age should also try to complement with other allergy information data, since it is uncertain how good dispensed allergy medication is as a proxy since it can be bought over the counter. Using complementary measures of allergy would also be useful for result comparison, since few countries have national medication registry data, which limits the number of countries which can reproduce the present study design.

## Conclusions

There was no increased risk of ever dispense of prescribed allergy medication among children 0–6 years, growing up close to a road with high traffic intensity, or high levels of NO_X_. These results may indicate that traffic-related exposure is not a risk factor for early onset allergy in children in southern Sweden. However, children with dispense of prescribed allergy medication may be a selected subgroup, and the results for this group may not be generalizable to all children with allergy.

## Additional file

Additional file 1: Table S1. Sensitivity analysis 1. Unadjusted HR (95 % CI) for allergy medication, in relation to traffic-related exposure, *n* = 26 128. **Table S2.** Sensitivity analysis 2. Malmo vs outside. Adjusted HR (95 % CI) for allergy medication, children in Malmö vs. outside Malmö. **Table S3.** Sensitivity analysis of children with high socio-economic status^a^, *n* = 3464. Adjusted^b^ HR (95 % CI) for allergy medication, in relation to traffic-related exposure. **Table S4.** Sensitivity analysis 4. Robustness of estimates in fully adjusted model, and model fit. Fully adjusted model including all possibly relevant covariates. HR (95 % CI) for allergy medication, in relation to traffic-related exposure, *n* = 5736. (DOCX 39.9 kb)
