# Supplementary material for: Ever dispense of prescribed allergy medication in children growing up close to traffic: a registry-based birth cohort
Source: BMC Public Health. 2015 Oct 6;15:1023. doi: 10.1186/s12889-015-2356-3 (PMC4595113; doi:10.1186/s12889-015-2356-3)
Supplement: Additional file 1: Table S1. — Sensitivity analysis 1. Unadjusted HR (95 % CI) for allergy medication, in relation to traffic-related exposure, n = 26 128. Table S2. Sensitivity analysis 2. Malmo vs outside. Adjusted HR (95 % CI) for allergy medication, children in Malmö vs. outside Malmö. Table S3. Sensitivity analysis of children with high socio-economic statusa, n = 3464. Adjustedb HR (95 % CI) for allergy medication, in relation to traffic-related exposure. Table S4. Sensitivity analysis 4. Robustness of estimates in fully adjusted model, and model fit. Fully adjusted model including all possibly relevant covariates. HR (95 % CI) for allergy medication, in relation to traffic-related exposure, n = 5736. (DOCX 39.9 kb) [file 12889_2015_2356_MOESM1_ESM.docx]

**Table S1. Sensitivity analysis 1.**

Unadjusted HR (95% CI) for allergy medication, in relation to traffic-related exposure, n=26 128

|  | Cases/total | Nasal anti-allergics  1st purchase | Cases/total | Oral antihistamine  1st purchase |
| --- | --- | --- | --- | --- |
| **Heaviest road ≤100m, birth address**  0-8639 cars/day  ≥8640 | 243/19523  86/6605 | 1.0  0.93(0.73-1.19) | 3996/19523  1349/6605 | 1.0  0.94(0.88-1.00) |
| **Heaviest road ≤100m, never moved**  0-8639 cars/day  ≥8640 | 183/19523  47/6605 | 1.0  0.80(0.58-1.10) | 3243/19523  950/6605 | 1.0  0.90(0.84- 0.97) |
| **NO_X,_ birth address**  ≤15 μg/m^3^  15-25  >25 | 101/7322  182/14642  44/4141 | 1.0  0.93(0.73-1.19)  0.88(0.62-1.26) | 1844/7322  2879/14642  616/4141 | 1.0  0.78(0.74-0.83)  0.74(0.67-0.81) |
| **NO_X_, never moved**  ≤15 μg/m^3^  15-25  >25 | 85/7322  123/14642  22/4141 | 1.0  0.96(0.73-1.27) 0.90(0.56-1.44) | 1595/7322  2187/14642  409/4141 | 1.0  0.78(0.74-0.84)  0.70(0.63-0.78) |
| **NO_X,_ lifetime mean**  ≤15 μg/m^3^  15-25  >25 | 86/5401  174/10831  20/2183 | 1.0  1.08(0.83-1.39)  0.85(0.52-1.38) | 1643/5471  2513/10917  359/2157 | 1.0  0.77(0.72-0.82)  0.63(0.56-0.70) |

**Table S2. Sensitivity analysis 2. Malmo vs outside.**

Adjusted HR (95% CI) for allergy medication, children in Malmö vs. outside Malmö.

|  | MALMÖ | | OUTSIDE MALMÖ | |
| --- | --- | --- | --- | --- |
|  | Nasal anti-allergics  1st purchase | Oral antihistamine  1st purchase | Nasal anti-allergics  1st purchase | Oral antihistamine  1st purchase |
| **Heaviest road ≤100m, birth address ^a^**  0-8639 cars/day  ≥8640 | 1.0  0.94(0.62-1.42) | 1.0  0.99(0.88-1.12) | 1.0  0.67(0.25-1.78) | 1.0  0.99(0.76-1.29) |
| **Heaviest road ≤100m, never moved ^a^**  0-8639 cars/day  ≥8640 | 1.0  0.85(0.48-1.50) | 1.0  0.92(0.79-1.06) | 1.0  0.65(0.21-1.98) | 1.0  1.00(0.73-1.36) |
| **NO_X,_ birth address ^a^**  ≤15 μg/m^3^  15-25  >25 | 1.0  1.16(0.68-1.96)  1.31(0.66-2.60) | 1.0  0.91(0.80-1.05)  0.88(0.72-1.08) | 1.0  0.55(0.16-1.83)  --- . | 1.0  0.97(0.75-1.27) 0.66(0.36-1.21) |
| **NO_X_, never moved ^a^**  ≤15 μg/m^3^  15-25  >25 | 1.0  1.38(0.75-2.56) 1.59(0.61-4.14) | 1.0  0.92(0.79-1.07) 0.83(0.64-1.07) | 1.0  0.87(0.25-3.02)  --- | 1.0  1.02(0.75-1.38) 0.52(0.19-1.40) |
| **NO_X,_ lifetime mean ^a^**  ≤15 μg/m^3^  15-25  >25 | 1.0  1.54(0.84-2.80)  1.02(0.36-2.87) | 1.0  0.87(0.75-1.00) 0.70(0.54-0.92) | 1.0  0.38(0.09-1.63)  --- | 1.0  0.87(0.65-1.15) 0.88(0.39-1.99) |

^a^ Adjusted for sex, season, parental origin, year of birth, breastfeeding, and parental allergy

--- Too few individuals, numerically unstable results.

**Table S3. Sensitivity analysis of children with high socio-economic status^a^, n=3464**

Adjusted^b^ HR (95% CI) for allergy medication, in relation to traffic-related exposure**.**

|  | Nasal anti-allergics  1st purchase | Oral antihistamine  1st purchase |
| --- | --- | --- |
| **Heaviest road ≤100m, birth address**  0-8639 cars/day  ≥8640 | 1.0  0.56(0.31-0.99) | 1.0  0.92(0.79-1.07) |
| **Heaviest road ≤100m, never moved**  0-8639 cars/day  ≥8640 | 1.0  0.49(0.21-1.15) | 1.0  0.87(0.72-1.05) |
| **NO_X,_ birth address**  ≤15 μg/m^3^  15-25  >25 | 1.0  0.78(0.48-1.28)  0.63(0.29-1.39) | 1.0  0.87(0.755-1.00)  0.78(0.62-1.00) |
| **NO_X_, never moved**  ≤15 μg/m^3^  15-25  >25 | 1.0  0.82(0.45-1.50)  0.30(0.04-2.26) | 1.0  0.88(0.75-1.03)  0.67(0.48-0.94) |
| **NO_X,_ lifetime mean**  ≤15 μg/m^3^  15-25  >25 | 1.0  0.82(0.48-1.40)  --- | 1.0  0.83(0.71-0.96)  0.64(0.44-0.93) |

^a^ High socioeconomic status here defined as children with both parents born in Sweden,

at least one parent with >12 years education, and never problem to pay bills.

^b^ Adjusted for sex, season, year of birth, breastfeeding, and parental allergy.

--- Too few individuals, numerically unstable results.

**Table S4. Sensitivity analysis 4. Robustness of estimates in fully adjusted model, and model fit.**

Fully adjusted model including all possibly relevant covariates. HR (95% CI) for allergy medication, in relation to traffic-related exposure, n= 5736.

|  | Nasal anti-allergics  1st purchase | N ^a^ | Model fit (AIC) | | | Oral antihistamine  1st purchase | N ^a^ | Model fit (AIC) | | |
| --- | --- | --- | --- | --- | --- | --- | --- | --- | --- | --- |
|  |  |  | Full model ^b^ | Stepwise model ^c^ | Unadjusted  model ^d^ |  |  | Full model ^b^ | Stepwise model ^c^ | Unadjusted model ^d^ |
| **Heaviest road ≤100m, birth address**  0-8639 cars/day  ≥8640 | 1.0  0.91(0.62-1.33) | 5736 | 2317 | 2303 | 2313 | 1.0  0.97(0.87-1.08) | 5736 | 27707 | 27692 | 27724 |
| **Heaviest road ≤100m, never moved**  0-8639 cars/day  ≥8640 | 1.0  0.80(0.48-1.34) | 5736 | 1525 | 1508 | 1505 | 1.0  0.91(0.80-1.05) | 5736 | 20456 | 20441 | 20465 |
| **NO_X,_ birth address**  ≤15 μg/m^3^  15-25  >25 | 1.0  0.96(0.63-1.46)  1.00(0.53-1.90) | 5734 | 2319 | 2305 | 2314 | 1.0  0.84(0.74-0.94)  0.79(0.65-0.96) | 5734 | 27683 | 27668 | 27690 |
| **NO_X_, never moved**  ≤15 μg/m^3^  15-25  >25 | 1.0  1.05(0.65-1.69)  0.97(0.37-2.53) | 5734 | 1528 | 1511 | 1508 | 1.0  0.87(0.76-0.99)  0.76(0.59-0.98) | 5734 | 20453 | 20437 | 20454 |
| **NO_X,_ lifetime mean**  ≤15 μg/m^3^  15-25  >25 | 1.0  1.06(0.70-1.62)  0.61(0.21-1.76) | 4958 | 1919 | 1905 | 1913 | 1.0  0.81(0.72-0.91)  0.64(0.49-0.83) | 5006 | 24427 | 24412 | 24425 |

^a^ Number of persons with complete outcome-, covariate-, and exposure information.

^b^Adjusted for sex, birth weight, smoking during pregnancy, environmental tobacco smoke (ETS), mold at home, parental

allergy, furred pets at home, breastfeeding, parental origin, parental education, problems to

pay bills, type of housing, season, and birth year.

^c^ Adjusted for sex, season, parental origin, year of birth, breastfeeding, and parental allergy.

^d^ Unadjusted model, only including exposure and outcome data.
